# Recoil Cavity Formation and Collapse for Drop Impact on Sieves

**DOI:** 10.1002/smsc.202400586

**Published:** 2025-06-06

**Authors:** Chandantaru Dey Modak, Prosenjit Sen

**Affiliations:** ^1^ Centre for Nano Science and Engineering Indian Institute of Science Bangalore 560012 India

**Keywords:** cavity collapse, droplet impact, recoil cavity, sieve, superhydrophobic

## Abstract

The principle underpinning most printing technologies rely on is the formation and subsequent collapse of cavities to generate high‐speed jets or droplets. Traditional methods, such as the Worthington jet or bubble‐based cavity, utilize the collapse mechanism to give rise to a high‐speed liquid jet. In contrast to known cavity collapse processes, a distinct phenomenon occurring during droplet impact on a superhydrophobic sieve is reported. Herein, the collapse of the impact cavity causes an air jet to rise through the sieve pore to form a “recoil cavity.” Subsequently, the recoil cavity collapses to eject a jet (droplets). The notable discovery is the emergence of the recoil cavity as a result of the impact cavity's collapse, which has been absent on any other surfaces. The present research explores the underlying mechanism and develops a model of the phenomenon. It is found that the process follows the principle of energy conservation, with a threshold energy flux ratio between impact and recoil driving the ejection of a single drop. These findings provide valuable insights for understanding drop impact printing techniques, which can be applied across various fields, including electronics, biology, and structural printing.

## Graphical abstract

A unique secondary cavity arises during droplet impact on a superhydrophobic sieve. Primary cavity collapse induces air suction through the sieve, forming a recoil cavity that collapses to eject droplets. A scaling model explains this process, revealing energy‐driven ejection dynamics and its implications on advanced printing in electronics, biology, and materials science.

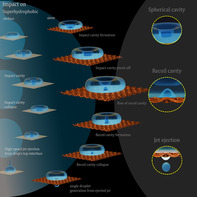

## 1 Introduction

The controlled generation of single droplets smaller than the capillary length ($l_{c}=\sqrt{\frac{\gamma }{\rho g}}$, where *γ* is surface tension, *g* is the acceleration due to gravity, and *ρ* is density) is of prime importance due to its broad applicability.^[^ ^1^, ^2^, ^3^ ^]^ Generation techniques work by focusing energy at the air–liquid interface to enable droplet separation against the capillary forces. Recent work^[^ ^1^ ^]^ has demonstrated the application of droplet impact on superhydrophobic sieves to generate tiny droplets. When a drop impacts a sieve, a jet of liquid penetrates the sieve only if the dynamic pressure ($\text{≈}\rho v^{2}$, where *v* is the impact velocity) exceeds the penetration pressure ($\text{≈}\frac{2\gamma }{L}$, where *L* is the pore opening of the sieve). Droplets can be generated from the ejected jet if its length ($l_{j}$) satisfies the Rayleigh criteria ($\frac{lj}{L}>\pi$). Interestingly, previous works^[^ ^4^, ^5^ ^]^ have shown single drop ejection in an impact regime where the penetration criterion was satisfied, but the ejection criterion was not. Droplet generation was enabled by the formation of a cavity and its collapse. The cavity collapse resulted in energy focusing and subsequent generation of the single droplet during the recoil phase.

**Figure 1:**
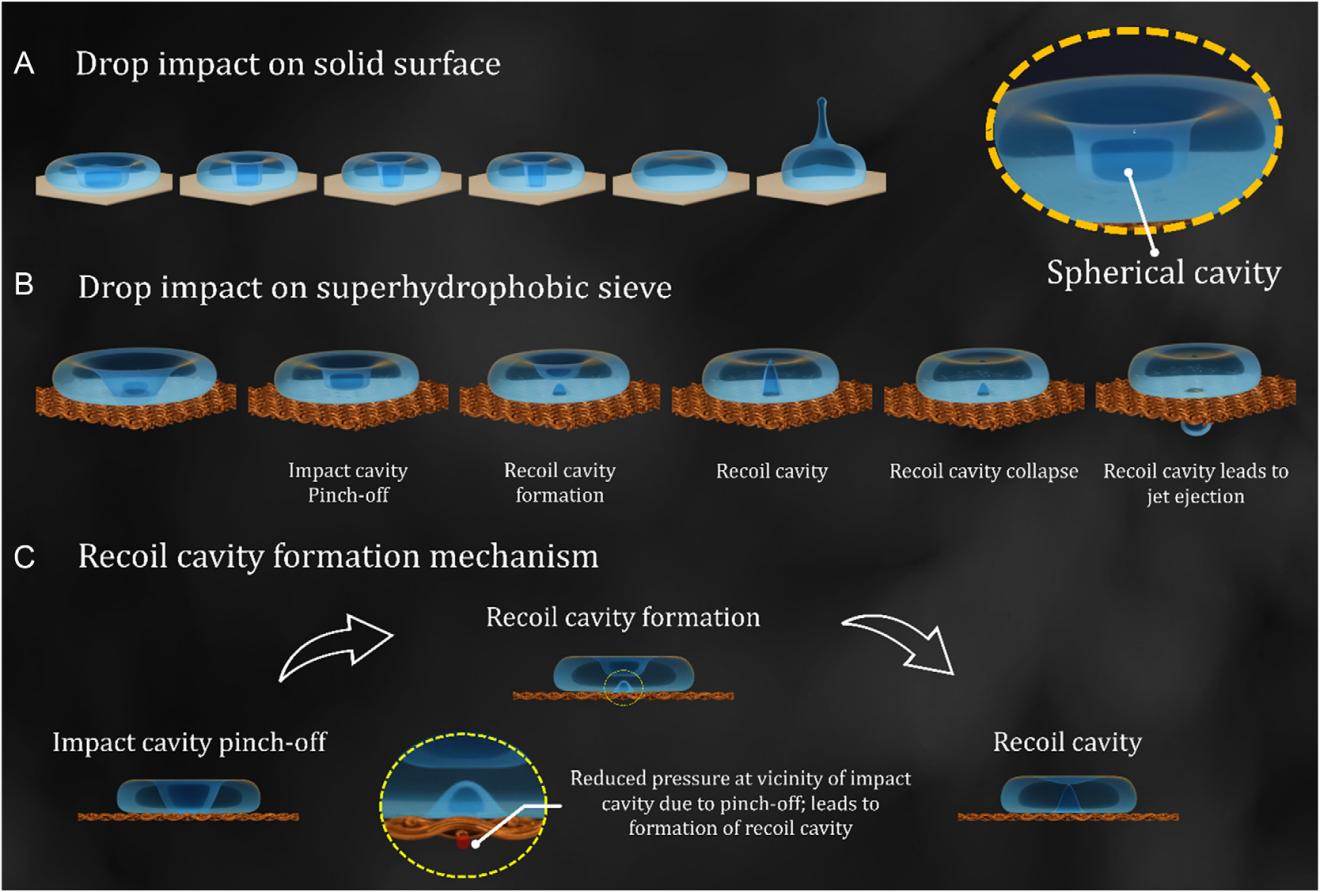
Formation and collapse of a recoil cavity for impact on a superhydrophobic mesh. A) Schematic of cavity dynamics for drop impact over the superhydrophobic surface. Collapse of the impact cavity drives a jet from the top. B) For drop impact on a superhydrophobic sieve, the impact cavity pinch‐off drives the formation of the recoil cavity. The collapse of the recoil cavity leads to a single droplet ejection. C) Schematic showing the mechanism of recoil cavity formation.

The formation of transient cavities and their singular collapse has been a matter of attention for decades.^[^ ^6^, ^7^, ^8^, ^9^, ^10^, ^11^ ^]^ Liquid jet emerging from the collapse has been observed under different scenarios such as droplet impact,^[^ ^10^ ^]^ the collapse of Faraday waves,^[^ ^11^ ^]^ cavitation bubble,^[^ ^12^ ^]^ deep pool impact,^[^ ^6^ ^]^ or bubble bursting at free interface.^[^ ^13^ ^]^ For impact on flat superhydrophobic surfaces, during the spreading phase, a cavity is formed at the center of the flattened droplet^[^ ^10^ ^]^ (see **Figure** **1**a). Impact‐induced capillary waves emerging from the spreading contact line converge at the droplet top to form the cylindrical air cavity.^[^ ^10^ ^]^ During droplet retraction, the cavity collapses. Driven by surface energy, the cavity interface velocity increases with time ($\propto \frac{1}{{\left(t_{0}-t\right)}^{0.5}}$, where $t_{0}$ is the collapse time). The momentum diverges, leading to a singularity at collapse.^[^ ^11^, ^12^ ^]^

Different collapse scenarios have been observed depending on the impact velocity.^[^ ^10^, ^14^, ^15^ ^]^ At lower impact velocities, the cavity remains cylindrical till it suddenly disappears. This is termed symmetric collapse. At higher impact velocities, the cylindrical column can break before the collapse. This breaking of the cylindrical column is called pinch‐off and leads to an asymmetric cavity collapse. Depending on the impact velocity, the cylindrical column can pinch off at different locations. On solid surfaces,^[^ ^10^ ^]^ pinch‐off from the top was reported at intermediate impact velocities. The top pinch‐off resulted in bubble entrapment. At higher impact velocities, a bottom pinch‐off was observed.

Usually, cavity formation and collapse are observed for a narrow range of impact velocities.^[^ ^11^, ^16^ ^]^ Cavity collapse with similar outcomes has been reported for surfaces with different wettability.^[^ ^14^, ^17^, ^18^ ^]^ Despite various experimental scenarios, only a single cavity formation has been reported in all droplet impact experiments. An interesting secondary cavity formation has been observed for impact on superhydrophobic sieves (meshes). As this secondary cavity is formed during the recoil phase of the droplet, it is termed the recoil cavity. This work investigates the properties and dynamics of the recoil cavity and its collapse. We further study the role of recoil cavity collapse in single droplet generation.

## 2 Results

### 2.1 Recoil Cavity Formation for Impact on a Sieve

10% and 30% (volume/volume) glycerol‐water (GW) mixture was used for the experiments. The higher viscosity of these solutions reduced the surface perturbation and improved cavity visibility. Experiments were performed on different pore‐opening sieves (Table S1, Supporting Information). A 2.55 mm drop was used. The impact height varied from 10 to 60 mm. The videos were captured using a high‐speed camera (Photron FastCam). The detailed imagining setup and the measured parameters are explained in Supplementary Figure S1, Supporting Information. The analysis was performed using MATLAB code and ImageJ software.

Unlike solid surfaces, we observe a distinctly different interface evolution for drop impact on a superhydrophobic sieve. In the initial part of the impact, the cavity formation process was similar to solid surfaces (Figure 1b(i–ii)). Capillary waves of wavelength ($\lambda \approx \frac{\gamma }{\rho v^{2}}$) were generated on impact. They propagate toward the drop center to form the impact cavity. The impact cavity pinches off from the bottom and collapses during droplet recoil. However, after the pinch‐off, a recoil cavity is formed from the droplet‐sieve interface (Figure 1b(iii–iv)). While the impact cavity collapses toward the top, the liquid interface deforms, and a jet of air rises through the sieve pore to form the recoil cavity (Figure 1c). This is absent for impact on solid surfaces. Subsequently, the recoil cavity collapses, ejecting a single drop (Figure 1b(v–vi)). Two questions arise from here. First, what drives the formation of the recoil cavity? Second, what leads to the ejection of a single droplet in a regime where the impact energy is insufficient to satisfy the ejection criteria? These questions are essential to understand as one drives the other.

### 2.2 What Drives Recoil Cavity Formation?

**Figure 2:**
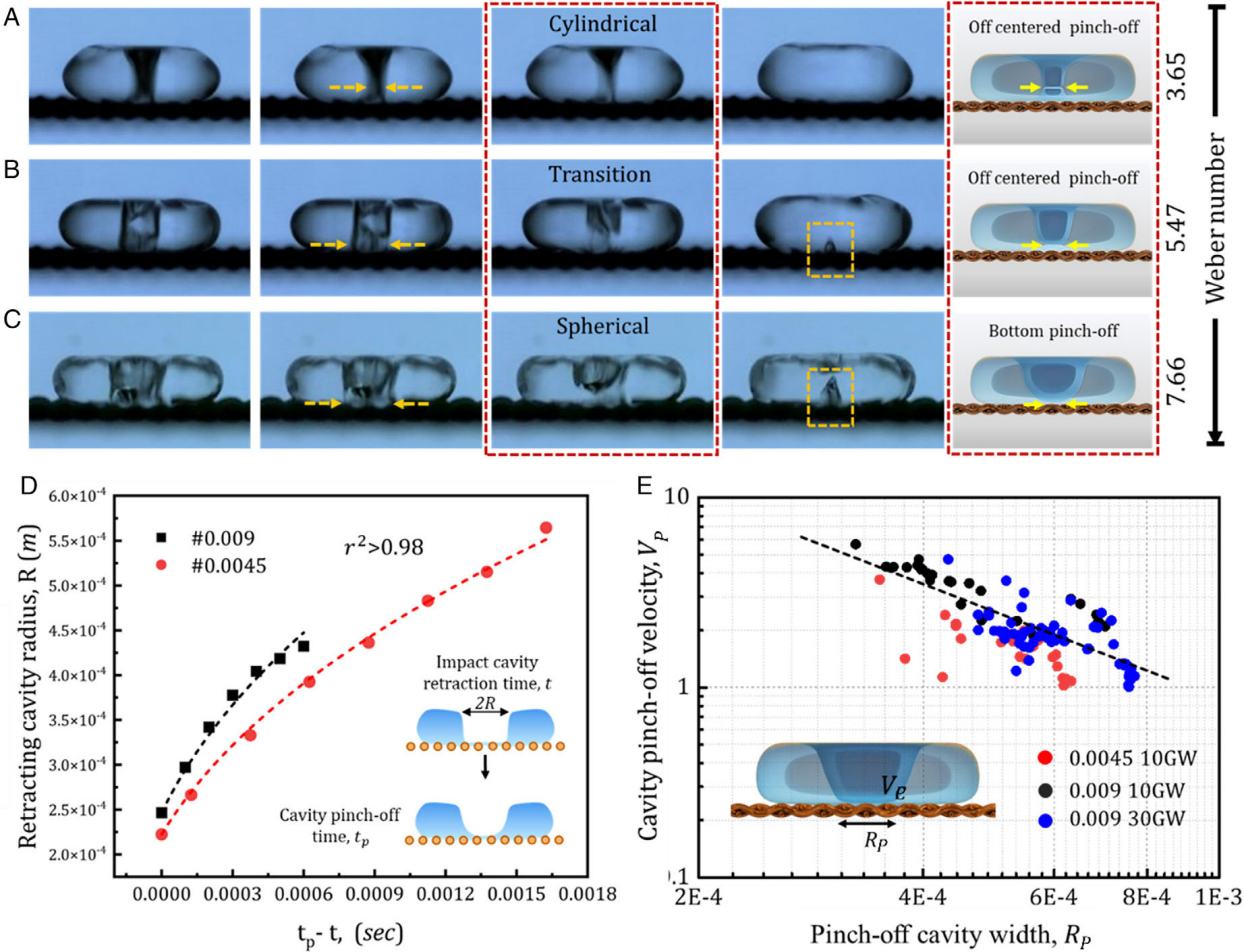
Evolution of cavity dynamics for: A) We = 3.65; B) We = 5.47; and C) We = 7.66. Schematic in the red dotted box shows the transition of the pinch‐off cavity from cylindrical to spherical shape with increase in Weber number. The impact cavity pinch‐off dynamics in terms of D) retraction cavity width with time for two sieves with different pore openings. The fitted α for #0.009 and #0.0045 sieve are 0.41 and 0.42 respectively. E) Impact cavity pinch‐off velocity versus pinch‐off cavity width for different pore openings and liquid viscosities. Viscous forces affect the dynamics of recoil cavity formation and collapse. At significantly high viscosities (>5 mPas), the formation of recoil cavities is suppressed. In the regime of viscosities included in the study (1.03 mPas for 10 GW and 1.8 for 30 GW), the dynamics of recoil cavity formation and collapse are primarily explained by inertial and capillary forces. Viscosity can be neglected without affecting the analysis or the conclusion.

The pinch‐off and the collapse of the cavity were analyzed frame‐by‐frame. The shape of the collapsing cavity changes with the Weber number (**Figure** **2**a–c). The cavity remains cylindrical at the lower Weber number (We < 4, mesh #0.009). As shown in Figure 2a, the cylindrical cavity pinches off at the middle of the droplet height. In this regime, the interface dynamics are similar to the droplet impact on solid surfaces. With a further increase in Weber number (We > 5.47, mesh #0.009), the pinch‐off position changes from the center to the bottom (Figure 2b,c). We also observe that the shape of the collapsing impact cavity changes from cylindrical to spherical (Figure 2a–c, schematic inset). In this regime (We > 7.3), recoil cavity formation is observed (Figure S2, Supporting Information). Recoil cavity was observed over a narrow range of We number (i.e., 7.3–13.5) for sieve #0.009. Beyond We > 15, the impact‐jet ejection is significant. The impact‐jet ejection suppresses the recoil cavity formation.

In the regime of recoil cavity formation, the evolution of the impact cavity shape on meshes is different from solid surfaces. After detachment from the bottom surface, the impact cavity assumes an approximately spherical shape. The dynamics of the impact cavity near pinch‐off are modeled using Rayleigh–Plesset (RP) equation.^[^ ^10^, ^14^, ^15^ ^]^ Due to the spherical evolution near pinch‐off, the RP equation for spherical free flow is used.^[^ ^19^ ^]^ Assuming the flow to be inviscid, varying radially and purely inertial, the equation reduces to

(1) $$R\ddot{R}+\frac{3}{2}{\left(\dot{R}\right)}^{2}=0$$

where *R*(*t*) is the radius of the spherical interface. On integrating with respect to time, the cavity radius, *R*(*t*) scales as ${\left[{\left(\frac{\gamma R_{0}^{2}}{\rho }\right)}^{0.5}\left(t-t_{p}\right)+R_{p}^{2.5}\right]}^{0.4}$ (detailed derivation in Supplementary file). Here, $R_{p}$ is the cavity radius at pinch‐off, and $t_{p}$ is the pinch‐off time. Figure 2d shows the plot of retracting cavity radius as a function of time $\left(t_{p}-t\right)$ for two sieves with pore openings. Our data indicates that the cavity radius follows a power law with an exponent of ≈0.4 near the pinch‐off, which agrees with our scaling. In contrast, the reported exponent is ≈0.5 for impacts on solid surface.^[^ ^10^ ^]^ Figure S3a, Supporting Information, plots the time evolution of the cavity radius near pinch‐off for different Weber numbers. Fits show the exponent *α* for different impact Weber numbers. As seen in Figure S3b, Supporting Information, there are two distinct regimes for *α*. At the lower Weber number (We ≈4), power *α* is 0.5, which relates to a cylindrical cavity collapse as seen for impact on solid surfaces. At higher Weber numbers (We ≥ 7.3), the power changed to 0.4, corresponding to spherical cavity dynamics. Hence, as the Weber number increases, the impact cavity collapse on meshes transitions from a cylindrical to a spherical shape.

The pinch‐off velocity ($V_{p}$) as a function of impact cavity width ($R_{p}$) can be obtained by differentiating R(t) with respect to time. This leads to

(2) $$V_{p}=0.4*{\left(\gamma R_{0}^{2}/\rho \right)}^{1/2}{\left(R_{p}\right)}^{-3/2}$$

Average velocity is measured by calculating the change in *R* over a fixed time (0.21 ms) close to the pinch‐off point. Fitting the data with an exponent of −1.5 shows good agreement with the experimental data (Figure 2e). This also supports the spherical shape of the collapsing impact cavity. The fitting is done in the regime of no‐drop and single‐drop ejection. This was due to higher interfacial capillary disturbance in the multiple droplet ejection regime which interferes with the measurement.

**Figure 3:**
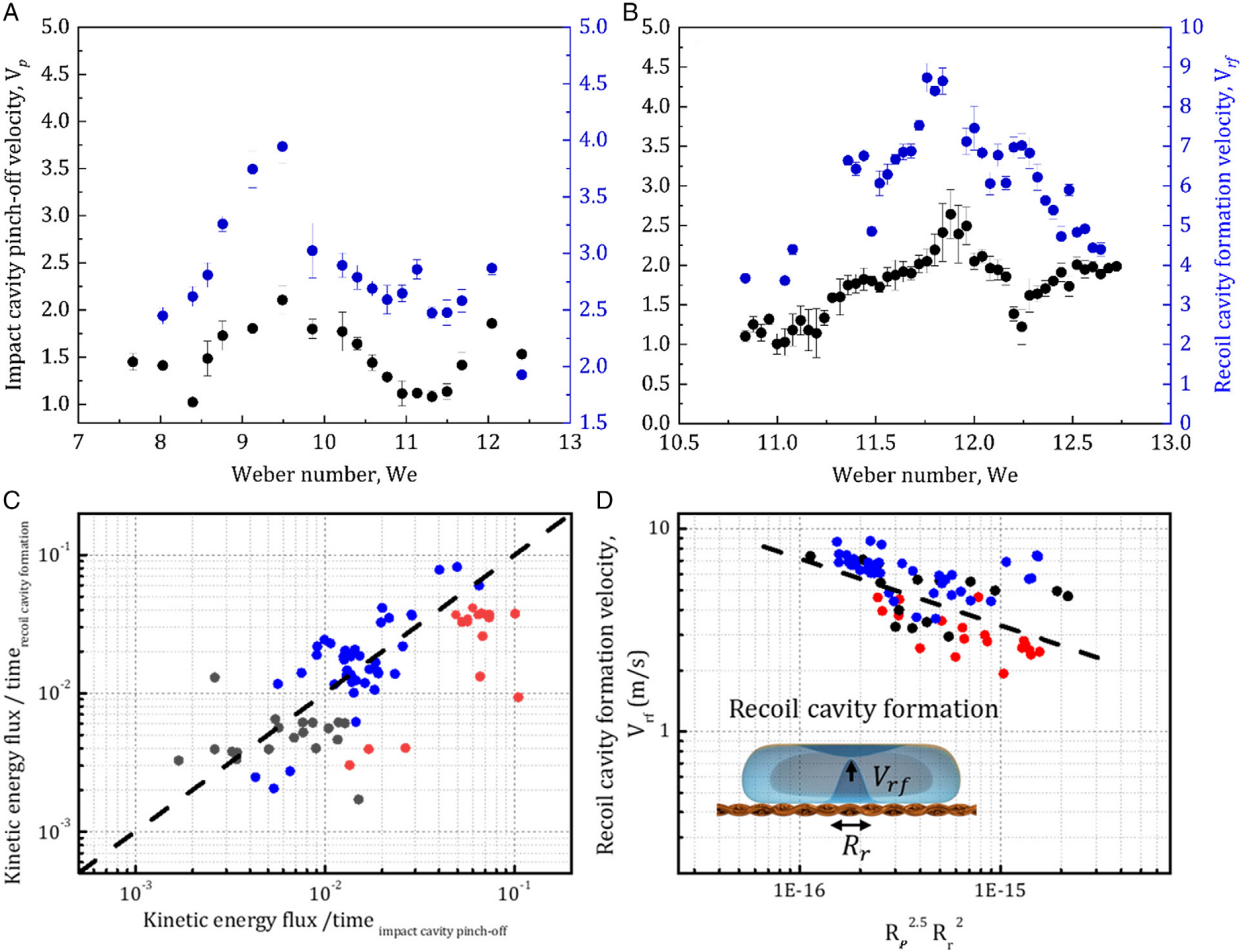
Plot of cavity velocities with Weber numbers A) sieve #0.009% and 10% glycerol water solution and B) sieve #0.009% and 30% glycerol water solution. The black and blue colors represent impact cavity pinch‐off velocity and recoil cavity formation velocity respectively. C) Plot of rate of change of kinetic energy fluxes between impact cavity pinch‐off and recoil cavity formation for different pore openings and viscosities. The black dotted line shows the scaling Equation (3). D) Plot between recoil cavity formation velocity versus radius factor ($R_{p}^{2.5}R_{r}^{2}$). The black dotted line in *D* shows the fitted scaling Equation (4).

The impact velocity is insufficient in the recoil cavity regime (We < 15) to generate a droplet from the impact jet. The impact jet rebounds. We first consider if the impact jet rebound is responsible for forming the recoil cavity. The capillary timescale ($\text{≈}{\left(\frac{\rho L^{3}}{\gamma }\right)}^{1/2}$) associated with the recoiling interface (≈0.5 ms, for sieve #0.009) is significantly smaller than the droplet spread time (≈3.3 ms). The penetrated jet retracts even before the droplet reaches its maximum spread diameter. Hence, retraction of the impact jet cannot explain the formation of the cavity observed during the recoil phase. Further, the retraction velocity of the impact jet (≈0.1–0.7 m s^−1^) is significantly smaller than the velocity with which the interface moves during the formation of the recoil cavity (≈>2 m s^−1^) as seen in **Figure** **3**a. Therefore, the inertial overshoot of the retracting impact jet cannot explain the recoil cavity formation.

The recoil cavity is formed only after the impact cavity pinch‐off. Hence, the interface dynamics after pinch‐off drives the recoil cavity formation. To understand the relationship between the two cavities, we plot their interface velocities for different Weber numbers and viscosity. For the Weber number range (7<We<9.5 for #0.009, 10% Glycerol Water solution), both impact cavity pinch‐off velocity and recoil cavity formation velocity increase (Figure 3a). Both velocities reach a maximum at a given Weber number. After that, both velocities decrease with Weber number. The same trend was observed for other sieves and liquid viscosities (Figure 3b, Figure S4, Supporting Information). This indicates the recoil cavity formation is driven by pinch‐off and subsequent collapse^[^ ^7^ ^]^ of the impact cavity.

The impact‐cavity pinch‐off and collapse can lead to a sudden localized reduction in liquid pressure^[^ ^10^, ^11^ ^]^ in the vicinity of the pinch‐off region (Video S1, Supporting Information). This leads to the suction of a jet of air, that is, growth of the recoil cavity (Figure 1c). To validate this theory, we applied the Bernoulli Equation at points 1 and 2 and then at points 2′ and 3 as shown in inset Figure S5, Supporting Information (sieve #0.009). The pressure reduction due to impact cavity pinch‐off can be calculated from points 1 and 2, whereas the required pressure to form the recoil cavity comes from points 2′ and 3. The pressure reduction or suction pressure is calculated as *P*_suc_ ≈ *P*_atm_ – K.E. where *P*_atm_ is atmospheric pressure and K.E. is the associated kinetic energies as shown in Figure S5, Supporting Information. Here, the Laplace pressure components are neglected due to their negligible contribution (≈5–10 times smaller than the kinetic components). The plot between cavity suction pressure ($P_{\text{suc}}$) with Weber number shows a drop in pressure at liquid–air interface and both the impact and recoil pressure are of the same order in magnitude. This sudden drop in pressure due to impact cavity pinch‐off is responsible for air suction and thus explains the formation of recoil cavity. This physics holds true for small pore opening sieves (139–279 μm) and liquid of low viscosities (1–2.5 mPas). However, for higher pore opening or higher viscosities, the impact jet retraction timescale is larger than cavity pinch‐off time, so the suction is not possible due to the hanging liquid jet (Figure S6, Video S2, Supporting Information).

### 2.3 Recoil Cavity Model

The recoil cavity grows from the bottom and rises toward the top interface of the drop. To model the formation of recoil cavity, we adopted the cavity flux equation from the literature^[^ ^10^, ^14^, ^15^ ^]^ based on kinetic energy considerations. The kinetic energies are an order of magnitude (≈10–100 times) greater than surface energies for both impact and recoil cavity. Hence, the surface energy term can be neglected in this case (Figure S7, Supporting Information). So, balancing the kinetic energy flux terms (Figure 3c), we get

(3) $$\frac{d}{dt}\left(\frac{1}{2}\rho V_{p}^{2}\frac{4}{3}\pi R_{p}^{3}\right)=\frac{d}{dt}\left(\frac{1}{2}\rho V_{\text{rf}}^{2}\pi R_{r}^{2}h\right)$$

where $R_{p}$ is the impact cavity radius, $V_{\text{rf}}$ is the recoil cavity formation velocity, $R_{r}$ is the recoil cavity radius, and *h* is the recoil cavity height. We assume a cylindrical cavity formation for the recoil cavity. This is based on experimental observation. Here, the recoil cavity radius remains almost constant while the cavity height changes rapidly.

Equation (3) simplifies as $4V_{p}^{3}R_{p}^{2}=V_{\text{rf}}^{3}R_{r}^{2}$. Figure 3c shows the comparison between the theoretical model and the experimental data. The energy flux rate for impact cavity pinch‐off is observed to be proportional to the recoil cavity formation across many experimental conditions. Therefore, it can be argued that the impact cavity pinch‐off drives the recoil cavity formation. Further from Equation (3), we can obtain an expression for recoil cavity formation velocity by substituting impact pinch‐off velocity ($V_{p}$) from Equation (2)

(4) $$V_{\text{rf}}={\left(4\right)}^{1/3}0.4{\left(\gamma R_{0}^{2}/\rho \right)}^{1/2}/{\left(R_{p}^{5/2}R_{r}^{2}\right)}^{1/3}$$

As seen in Figure 3d, Equation (4) provides a good prediction for the scaling relations between $V_{\text{rf}}$, $R_{p}$, and $R_{r}$.

Thus far, we established that the impact cavity pinch‐off induces the recoil cavity. But, at a low Weber number (We < 7) regime, we observed that the impact cavity pinch‐off velocities are higher, and surprisingly, the recoil cavity was absent (Figure S8a, Supporting Information). This is due to the pinch‐off position of the cavity (Figure 2a–c). At lower Weber number experiments, we observed that the cavity pinch‐off position is close to the center of the drop height instead of at the bottom. This results in approximately symmetric flows driven toward the top and bottom which ejects out a jet while the drop recoils.

### 2.4 Single Drop Ejection in Recoil Cavity Collapse

**Figure 4:**
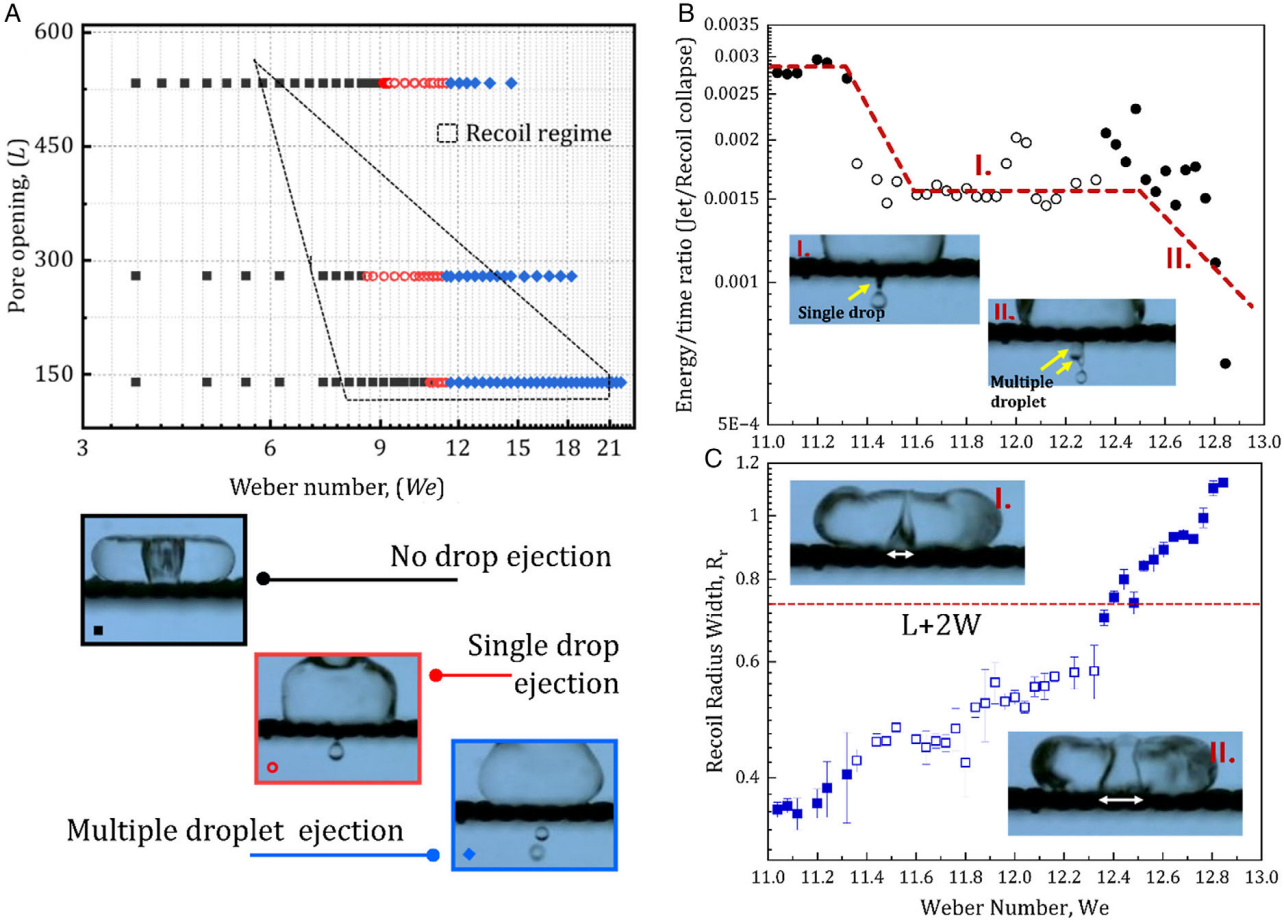
A) Phase diagram showing recoil cavity formation and single droplet ejection in terms of pore opening and Weber number for 10% glycerol water mixture. The black‐dotted region shows the recoil cavity formation region. Different color points depict the droplet ejection zone. Black color shows no drop ejection regime. Red color shows the single‐drop ejection regime. And the blue represents the multiple droplet ejection zone. The same has been represented in the images below. B) shows the ratio of energy fluxes per unit time between recoil jet and recoil cavity collapse with Weber number. C) shows recoil radius width with Weber number. (I) Ejection of single drop through #0.009 sieve and recoil cavity width and (II) ejection of multiple jet through sieve #0.009. The difference between two scenarios is in the ejection process. Sieve #0.009 eject single droplet due to smaller cavity width. While for multiple jets, recoil radius increases (exceeding *L* + 2 *W*, where *L* is sieve pore length and *W* is sieve wire diameter), causing energy focusing to extend beyond a single pore, resulting in multiple droplet ejections. The open symbols represent single‐drop ejection.

The recoil cavity collapses to eject out a jet, which in turn produces a single droplet. Single droplet ejection is observed for a specific Weber number range, as shown in **Figure** **4**a. A regime map for different pore openings with Weber numbers has been plotted. For sieve #0.009 (pore opening–279 μm) and 10% GW liquid, the single droplet is generated in the Weber number range of 8.5–11.3. In comparison, for smaller pore openings (sieve #0.0045), the single droplet is generated for a smaller Weber number range of 10.8–11.5. For both the sieves (#0.009, #0.00045), the single droplet ejection occurs in the recoil cavity collapse mode.

It is interesting to note that the formation of the recoil cavity is not sufficient condition for generating single droplets. The recoil jet formed due to recoil collapse will eject single or multiple drops depending on the collapse energy. To understand this, we plotted the ratio of energy fluxes per unit time between the ejected jet and recoil cavity collapse with Weber number (Figure 4b). This captures the rate of energy transfer to the jet from the collapse of the recoil cavity. The process of recoil cavity collapse and jet formation is shown in the schematic (Figure S1, Supporting Information). Interestingly, the ratio (energy flux/time) remains constant for a single drop ejection zone. This indicates an energy‐focusing mechanism that leads to a single drop.

At low Weber numbers, the energy flux is focused, and the ratio is higher. However, the jet ejection velocity is insufficient (*V*_j_ < 1.35 m s^−1^) to overcome the interfacial energy barrier (Figure S9, Supporting Information) for droplet ejection. A higher Weber number, due to an increase in recoil cavity width (exceeding *L* + 2 *W*, where *L* is sieve pore length and *W* is sieve wire diameter), causes energy focusing to extend beyond a single pore, resulting in multiple droplet ejections (Figure 4c). And finally, at a single droplet ejection zone, the jet ejection velocity^[^ ^20^ ^]^ is sufficient (>((8 × *γ*)/(*ρ* × *L*))^0.5^≈1.35 m s^−1^). The recoil cavity width increases with Weber number but remains smaller than the *L* + 2 *W*, so the energy flux of the jet is concentrated on the fixed pore and thus remains constant. This leads to a single drop at a certain Weber number range. Further, the threshold range of a single drop can be correlated with the theoretical model for number of ejected droplets.^[^ ^5^ ^]^ The model predicts the number of droplets ejected from a single jet for different jet velocities. For our case, the maximum critical velocity (≈1.785 m s^−1^) at transition point follows the model when computed for two drop ejections as shown in Figure S9, Supporting Information, which explains the extremes of single droplet range.

For smaller pore opening, the single droplet ejection starts at a higher Weber number (We > 10.8, #0.0045 sieve). This is due to the lower pore opening (≈139 μm) that requires higher collapse energy to eject. Additionally, the range of Weber numbers for single droplet ejection for sieve #0.0045 is lower than #0.009 (Figure 4a). This can be explained by considering the collapse energy distribution and the recoil cavity width. The recoil cavity radius in a single droplet regime for sieve #0.0045 is ≈2(*L* + *W*) whereas for sieve #0.009 it is less than (*L* + *W*) (Figure S8b, Supporting Information). Hence, for sieves with smaller pore openings, the collapse energy is distributed across multiple pores, thereby limiting the single drop regime.

### 2.5 Droplet Generation in High Pore Opening Sieves

The recoil dynamics and single droplet ejection were studied for high pore opening sieve (#0.012). The phenomena were different when compared to the lower pore opening sieves. We found a very small Weber number region around (≈6) where a recoil cavity is formed (Figure 4a) but collapse doesn't lead to single droplet generation. This is because the collapse energy is not sufficient to eject and give a single drop. Thereafter, only impact cavity formation and collapse were observed for all Weber numbers, and recoil cavity was absent (Figure 4a). This is attributed to the dynamics of the impact jet. Specifically for high pore opening meshes, beyond We > 7, the penetrated jet volume increases as the Weber number increases. As the impact jet volume increases, the jet retraction time also increases (Figure S10a, Supporting Information). Therefore, after We > 7, the impact cavity pinches off before the impact jet retracts. This prevents the formation of a recoil cavity as the suction pressure pulls back the liquid in the still‐hanging impact jet (Figure S6, Video S2, Supporting Information).

Further, for higher pore opening mesh, we found that the single drop ejection (9.1 < We < 11.5) occurs from the impact‐penetrated jet (Figure 4a). Beyond We > 11.5, multiple droplets eject from multiple penetrating jets. The impact cavity pinch‐off velocity first increases and then decreases (Figure S10b, Supporting Information) with Weber number. Single droplet ejection occurs from the hanging jet at the cavity pinch‐off velocity peak (We ≈9.1). At the maximum pinch‐off velocity, there is a sudden reduction in local pressure which can be calculated from the Bernoulli equation (Δ $P_{\text{drop}}\text{≈}0.5*\rho *V_{p}^{2}$≈4590 atm). The pressure drop decelerates (pulls back) the liquid in the impact jet. This deceleration will lead to the necking of the jet to eject a single droplet (Figure S10c, Supporting Information). Furthermore, from We > 9.1, we see a continuous decrease in cavity pinch‐off velocity, then what drives the single droplet ejection? So other than pinch‐off velocity, the jet's kinetic energy also contributes to the ejection phenomena. After the maximum peak, as the Weber number is increasing, the jet velocity increases. So, the jet's kinetic energy pulls the jet forward and the pinch‐off leads to necking. And finally, after We > 9.4, the impact penetration energy is sufficient enough to eject single drop (Figure S10d, Supporting Information) and thus eject droplet way before pinch‐off.

In summary, our investigation has centered on a novel phenomenon related to the collapse of cavities, specifically observed in sieve configurations. When a drop impacts a sieve‐structured surface, collapse of the conventional impact cavity triggers a sudden air suction at the base of the sieve‐drop interface, resulting in the formation of a recoil cavity. Within specific Weber number ranges, the collapse of this recoil cavity leads to the expulsion of individual droplets. The recoil cavity was derived by impact cavity pinch‐off depending on cavity shape and pinch‐off position. A scaling model was presented that explains the formation of the recoil cavity and single ejection zone. We identified two distinct modes of droplet ejection for sieves with different pore sizes and elucidated the underlying ejection mechanism. Ultimately, our research demonstrates its potential applicability in a range of printing applications, encompassing electronic, biological, and structural printing.

## 3 Experimental Section

3.1

3.1.1

##### Sieve Fabrication

The superhydrophobic sieve fabrication requires two basic steps, etching and surface coating. Etching is done using etchant sodium hydroxide (2.5 mol L^−1^) and ammonium persulphate (0.1 mol L^−1^). The sieve was dipped for 15 min in the etchant (in equal volume ratio). The etched sieve after 15 min was air dried and dipped in Teflon for 20 min. The coated sieve is further air dried and heated at 120 °C for 15 min. The nanowires formed have a height, of 10 μm, and a mean tip diameter of 180 nm. The static contact angle comes out to be 169° ± 4°.

## Conflict of Interest

The authors declare no conflict of interest.

## Author Contributions

**Chandantaru Dey Modak**: conceptualization (equal); data curation (equal); formal analysis (lead); investigation (lead); methodology (lead); validation (equal); writing—original draft (equal); writing—review and editing (equal). **Prosenjit Sen**: conceptualization (equal); data curation (equal); formal analysis (supporting); funding acquisition (lead); project administration (lead); resources (lead); supervision (lead); writing—original draft (equal); writing—review and editing (equal).

## Supporting information

Supplementary Material

## Data Availability

The data that support the findings of this study are available from the corresponding author upon reasonable request.

## References

[1] C. D. Modak , A. Kumar , A. Tripathy , P. Sen , Nat. Commun. 2020, 11, 4327. 32859927 10.1038/s41467-020-18103-6 PMC7455714

[2] R. Lathia , K. N. Nampoothiri , N. Sagar , S. Bansal , C. D. Modak , P. Sen , Langmuir 2023, 39, 2461. 36779356 10.1021/acs.langmuir.2c02905

[3] R. Lathia , S. Nagpal , C. D. Modak , S. Mishra , D. Sharma , B. S. Reddy , P. Nukala , R. Bhat , P. Sen , Nat Commun 2023, 14, 6445. 37833273 10.1038/s41467-023-41977-1 PMC10575970

[4] A. Kumar , A. Tripathy , C. D. Modak , P. Sen , J. Microelectromech. Syst. 2018, 27, 866.

[5] É. Lorenceau , D. Quéré , J. Colloid Interface Sci. 2003, 263, 244. 12804909 10.1016/s0021-9797(03)00126-7

[6] G.‐J. Michon , C. Josserand , T. Séon , Phys. Rev. Fluids 2017, 2, 23601. 10.1103/PhysRevLett.122.014501 31012665

[7] S. Gekle , J. M. Gordillo , J. Fluid Mech. 2010, 663, 293.

[8] Y. Lee , S. Shin , G. Choi , H. Jeon , Y. Kim , H. Kim , Phys. Fluids 2020, 32, 112104.

[9] S. T. Thoroddsen , K. Takehara , H. D. Nguyen , T. G. Etoh , J. Fluid Mech. 2018, 848, R3.

[10] D. Bartolo , C. Josserand , D. Bonn , Phys. Rev. Lett. 2006, 96, 124501. 16605909 10.1103/PhysRevLett.96.124501

[11] B. W. Zeff , B. Kleber , J. Fineberg , D. P. Lathrop , Nature 2000, 403, 401. 10667786 10.1038/35000151

[12] S. R. Gonzalez‐Avila , D. M. Nguyen , S. Arunachalam , E. M. Domingues , H. Mishra , C.‐D. Ohl , Sci. Adv. 2020, 6, eaax6192. 32258392 10.1126/sciadv.aax6192 PMC7101208

[13] L. Duchemin , S. Popinet , C. Josserand , S. Zaleski , Phys. Fluids 2002, 14, 3000.

[14] L. Chen , L. Li , Z. Li , K. Zhang , Langmuir 2017, 33, 7225. 28661691 10.1021/acs.langmuir.7b01506

[15] J. Guo , S. Zou , S. Lin , B. Zhao , X. Deng , L. Chen , Phys. Fluids 2020, 32, 122112.

[16] Z. Q. Yang , Y. S. Tian , S. T. Thoroddsen , J. Fluid Mech. 2020, 904, A19.

[17] Y. Renardy , S. Popinet , L. Duchemin , M. Renardy , S. Zaleski , C. Josserand , M. A. Drumright‐Clarke , D. Richard , C. Clanet , D. Quéré , J. Fluid Mech. 2003, 484, 69.

[18] K. Yamamoto , M. Motosuke , S. Ogata , Appl. Phys. Lett. 2018, 112, 1.

[19] P. Sleutel , Droplets: Drag, Coalescence and Impact, University of Twente, Enschede 2017, p. 127.

[20] P. C. Duineveld , M. M. De Kok , M. Buechel , A. Sempel , K. A. H. Mutsaers , P. Van de Weijer , I. G. J. Camps , T. Van de Biggelaar , J.‐E. J. M. Rubingh , E. I. Haskal , Proc. SPIE 4464, Organic Light-Emitting Materials and Devices V, SPIE, San Diego, CA 2002, pp. 59–67.

