# Supplementary material for: Recoil Cavity Formation and Collapse for Drop Impact on Sieves
Source: Small Sci. 2025 Jun 6;5(7):2400586. doi: 10.1002/smsc.202400586 (PMC12257895; doi:10.1002/smsc.202400586)
Supplement: Supplementary file 1 — Supplementary Material [file SMSC-5-2400586-s001.zip › smsc12729-sup-0001-SuppData-S1.pdf]

Supplementary Information for

Recoil Cavity Formation and Collapse for Drop Impact on Sieves

Chandantaru Dey Modak[1], and Prosenjit Sen[1*]

[1]Centre for Nano Science and Engineering, Indian Institute of Science, Bangalore, India, 560012

[*]**Corresponding Author's Email:** prosenjits@iisc.ac.in

This PDF file includes the following:

Supplementary text

Tables S1

Figs. S1 to S10

Captions for movies S1, S2

References for SI reference citations

Other supplementary materials for this manuscript include the following:

Movies S1, S2

Supplementary Information Text

Section 1: Impact cavity interface velocity

(A) For cylindrical cavity (We< 5.47)

Raleigh-Plesset equation for cylindrical evolution of the cavity assuming flow to be inertial and inviscid is given by

$$R\ddot{R} + (\dot{R})^2 = 0 \tag{1}$$

where $R(t)$ is the radius of the cavity. Now assuming a power law solution with time, we can write cavity $R(t)$ as

$$[R(t)]^{\frac{1}{\alpha}} = At + B \tag{2}$$

Substituting (2) in (1), we can evaluate α – 0.5. The value of α is calculated based on consistent asymptotic balance for power law equation (2).

Since we are interested in the dynamics of the impact cavity near pinch-off, we use the following boundary condition.

$R(t) = R_p$ at $t = t_p$

where R_p is the cavity radius at pinch-off and t_p is the pinch-off time. Substituting value of α – 0.5 and applying the boundary condition in equation (2)

$$[R(t)]^2 - [R_p]^2 = A(t - t_p) \tag{3}$$

Using dimensional analysis, we can estimate $A \propto \sqrt{\frac{\gamma R_0}{\rho}}$, where γ is the surface tension of the liquid, R_O is the radius of impacted drop, and ρ is the density of the liquid. The value of the

experimentally observed exponent (α) can vary depending on the nature of cavity pinch-off and the governing dynamics[1] as shown in section 1B for spherical cavity collapse. Further, to obtain an expression of impact cavity retraction velocity near pinch-off, equation (3) is differentiated to obtain

$$2\dot{R}R \sim \left(\frac{\gamma R_0}{\rho}\right)^{\frac{1}{2}} \qquad (4)$$

Rearranging the equation (4), the velocity of the impact cavity near pinch-off is given by

$$V_P \sim 0.5 \left(\frac{\gamma R_0}{\rho}\right)^{\frac{1}{2}} \left(R_p\right)^{-1} \quad \text{at} \quad R = R_p \qquad (5)$$

The above V_P is velocity of pinch-off for cylindrical cavity collapse dynamics (We< 5.47)

(B) for spherical cavity (We>5.47)

Raleigh-Plesset equation for spherical evolution of the cavity assuming flow to be inertial and inviscid is given by

$$R\ddot{R} + \frac{3}{2}(\dot{R})^2 = 0 \qquad (6)$$

where $R(t)$ is the radius of the cavity. Integrating the equation (6), we obtain a power law equation of cavity $R(t)$

$$[R(t)]^{\frac{1}{\alpha}} = At + B \qquad (7)$$

Substituting (7) in (6), we can evaluate $\alpha = 0.4$. Since we are interested in the dynamics of the impact cavity near pinch-off, we use the following boundary condition.

$R(t) = R_p$ at $t = t_p$

where R_p is the cavity radius at pinch-off and t_p is the pinch-off time. Substituting the value of α and boundary condition in equation (7) we get,

$$[R(t)]^{5/2} - [R_p]^{\frac{5}{2}} = A(t - t_p) \quad (8)$$

Using dimensional analysis, we can estimate $A \propto \sqrt{\frac{\gamma R_0^2}{\rho}}$, where γ is the surface tension of the liquid, R_O is the radius of impacted drop, and ρ is the density of the liquid. Further, to obtain an expression of impact cavity retraction velocity near pinch-off, equation (8) is differentiated to obtain

$$R^{\cdot} R^{\frac{3}{2}} \sim 0.4\left(\gamma \frac{R_O^2}{\rho}\right)^{\frac{1}{2}} \quad (9)$$

Rearranging the equation (9), the velocity of the impact cavity near pinch-off is given by

$$V_P \sim 0.4\left(\gamma \frac{R_O^2}{\rho}\right)^{\frac{1}{2}} (R_p)^{\frac{-3}{2}} \quad \text{at} \quad R = R_p \quad (10)$$

Section 2: Energy flux per unit time

Retraction of the impact cavity drives the formation of the recoil cavity. We argue that under this assumption, the energy flux should be conserved. In formulating the conservation equation, the surface energies of the cavities are neglected as the ratios of kinetic energy to surface energy is one order greater in magnitude. Then, the conservation of kinetic energy flux for the impact and recoil cavity leads to

$$\frac{d}{dt}\left(\frac{1}{2}\rho V_P^2 \frac{4}{3}\pi R_P^3\right) = \frac{d}{dt}\left(\frac{1}{2}\rho V_{rf}^2 \pi R_r^2 h\right) \quad (11)$$

Differentiating equation (11), we get

$$2V_P^3 R_P^2 = \frac{1}{2} V_{rf}^3 R_r^2 \tag{12}$$

Here, $V_P = \frac{dR_P}{dt}$ and $V_{rf} = \frac{dh}{dt}$.

Now, the expression for recoil cavity formation can be obtained by substituting the impact cavity collapse velocity, equation (10) in equation (12) for spherical cavity collapse.

$$V_{rf} = \frac{(4)^{\frac{1}{3}} 0.4 \left(\gamma \frac{R_O^2}{\rho}\right)^{{\frac{1}{2}}^{\frac{1}{3}}}}{\left(R_P^{\frac{5}{2}} R_r^2\right)} \tag{13}$$

Mesh Type/ Dimensions	Pore opening, *L* *Mm*	Wire Diameter, *W* μm	Solid fraction (ϕ)	Breakthrough pressure (Pa)
#0.0045	140.0	114.3	0.7	2.06×10^3
#0.009	279.0	228.60	0.7	1.03×10^3
#0.012	533.4	304.80	0.6	5.40×10^2

Liquid	Density, ρ kg/m^3	Surface tension, γ kg/s^2
10GW	1020.7	0.069934
30GW	1097.1	0.06835

Table S1: Mesh and liquid properties

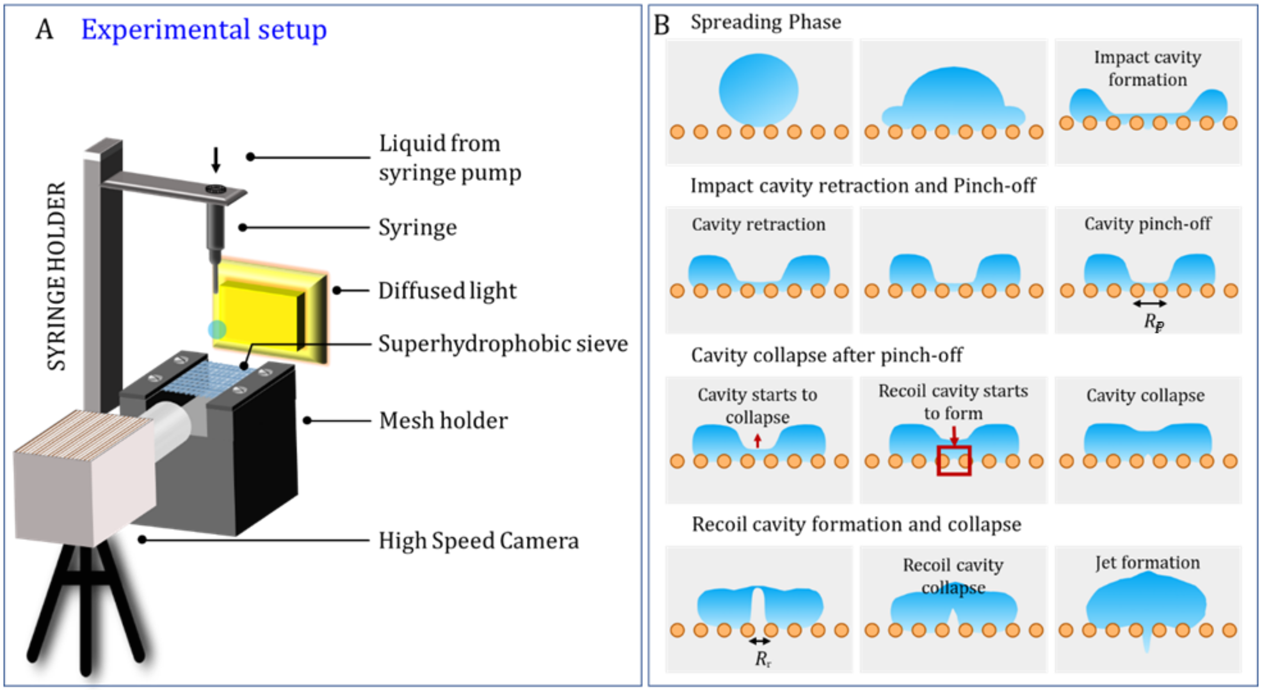

Figure S1: Experimental setup and Measurement Parameters (A) Experimental design showing different accessories used. (B) Various stages of drop impact on a superhydrophobic sieve. Measured parameters are shown.

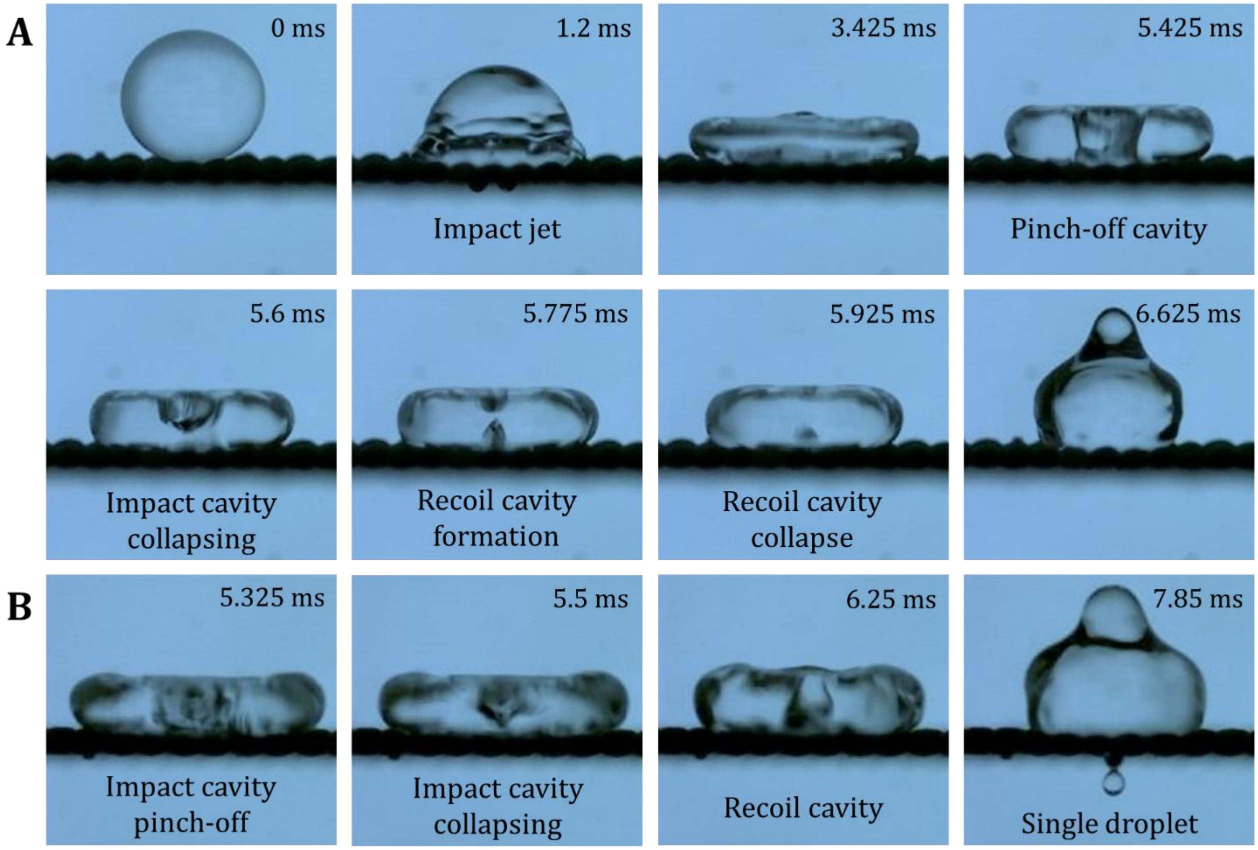

Figure S2: Recoil Cavity formation at different Weber number. (a) We ~ 7.6; (b) We ~ 10.94. Time sequence images show different cavity pinch-off stage. The spherical cavity pinch-off leads to formation of recoil cavity for We number range 7.3 < We < 13.5 for sieve #0.009 of pore opening 279μm and liquid 10 percent Glycerol Water solution. At We ~ 10.94, recoil cavity collapse leads to single droplet generation.

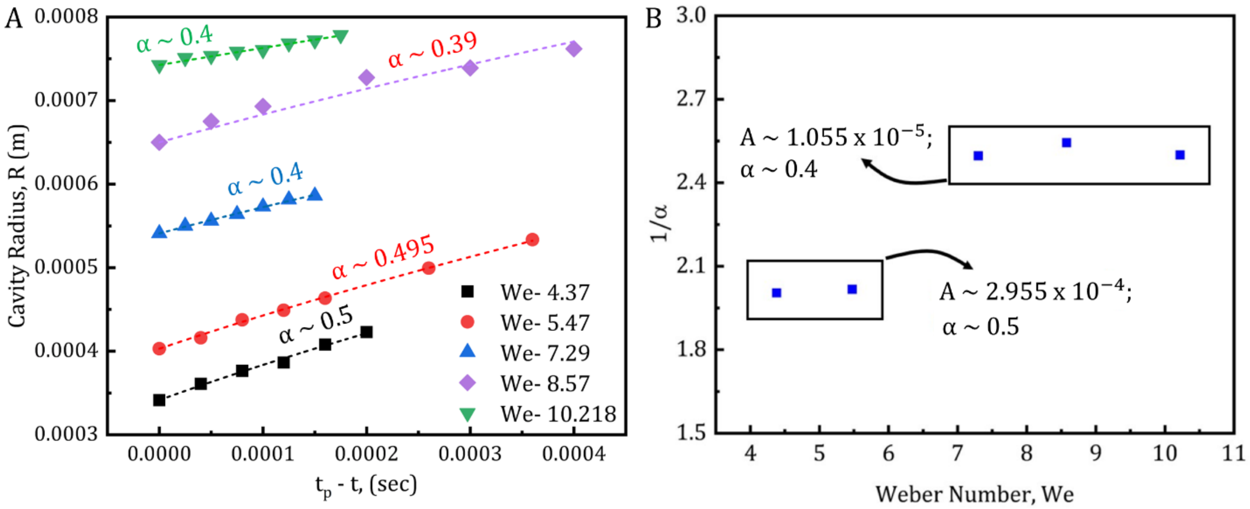

Figure S3: Power Law relation from Rayleigh-Plesset Equation. (A) The plot of cavity pinch-off radius, R versus $(t_p - t)$ for various Weber numbers. (B) The plot of $(\frac{1}{\alpha})$ versus Weber number shows sudden power change when the cavity dynamics change from cylindrical to spherical. The theoretical model $[R(t)]^{1/\alpha} = A(t - t_p) + [R_p]^{\frac{1}{\alpha}}$ is fitted by keeping the A as constant. For cylindrical cavity collapse, α is 0.5 and $A \propto \sqrt{\frac{\gamma R_0}{\rho}}$. For spherical cavity collapse α is 0.4 and A $\propto \sqrt{\frac{\gamma R_0^2}{\rho}}$.

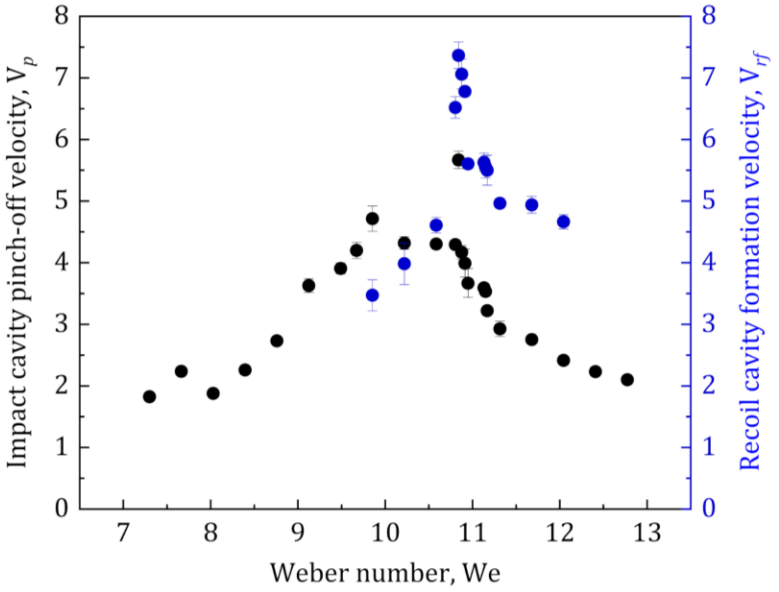

Figure S4: Plot between impact cavity pinch-off velocity and recoil cavity formation velocity versus Weber number for sieve #0.0045 with pore opening 139μm, 10 percent glycerol water solution.

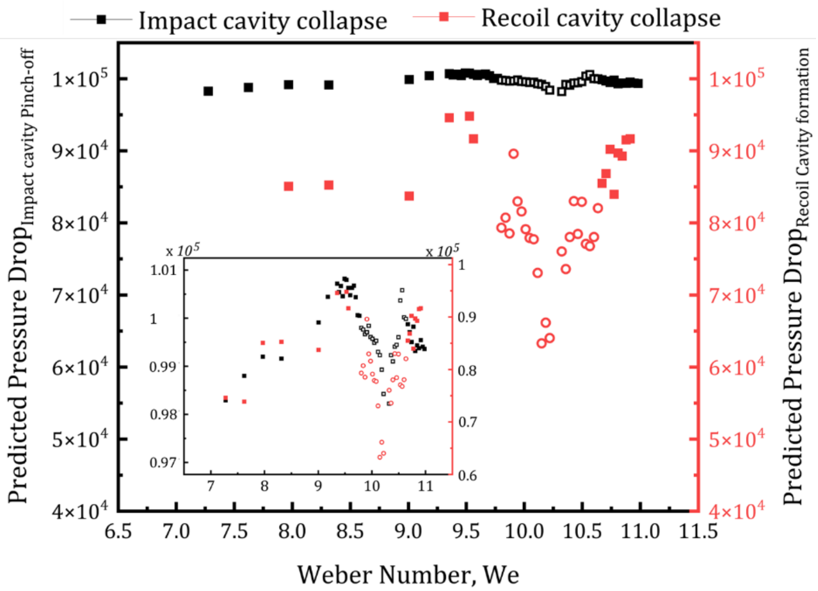

Figure S5: Plot showing suction pressure versus Weber number. Applying the Bernoulli equation at points 1 and 2 gives us the suction pressure created due to impact cavity pinch-off (indicated by black points). At 2' and 3, the plot predicts the required suction pressure to form a recoil cavity (indicated by red points). Both the pressures converge with the Weber number, showing that impact pinch-off drives the formation of the recoil cavity. Inset showing the points at the liquid-air interface at the sieve bottom where the Bernoulli Equation is applied. Here P_1 and P_3 are at atmospheric pressure. The open symbols represent a single drop ejection zone. Here, the Laplace pressure components are neglected due to negligible contributions. The inset shows the pressure drop plots in two different axes to show similar shape in pressure trend.

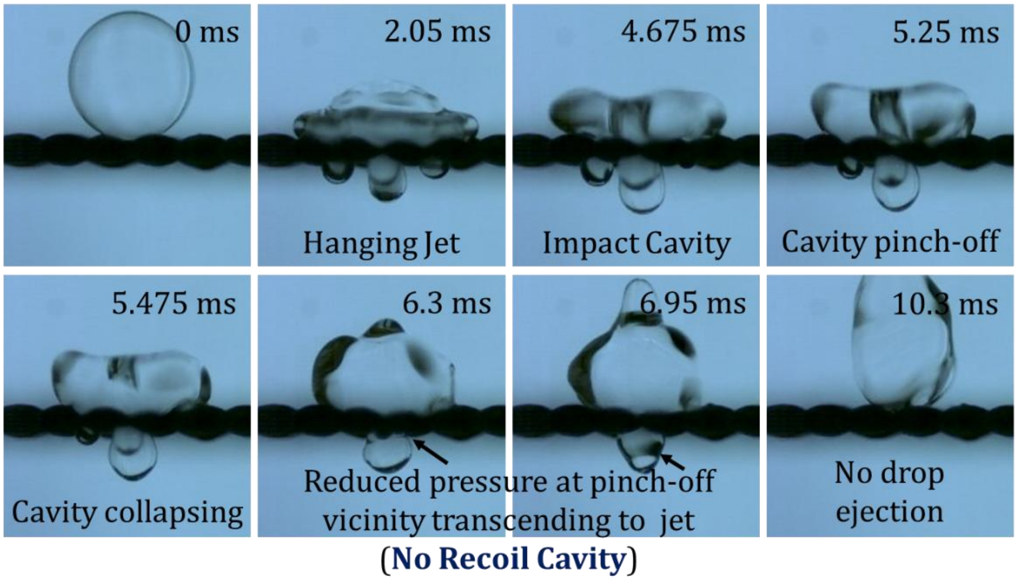

Figure S6: Time-lapse sequence of drop cavity dynamics for impact on a superhydrophobic sieve of pore opening 534 μm (Weber number – 8.95; 10% glycerol water solution). The penetrating impact-jet keeps hanging throughout the cavity collapse process, and due to this recoil cavity doesn't form. The jet retraction time is ~2 times greater than the pinch-off time.

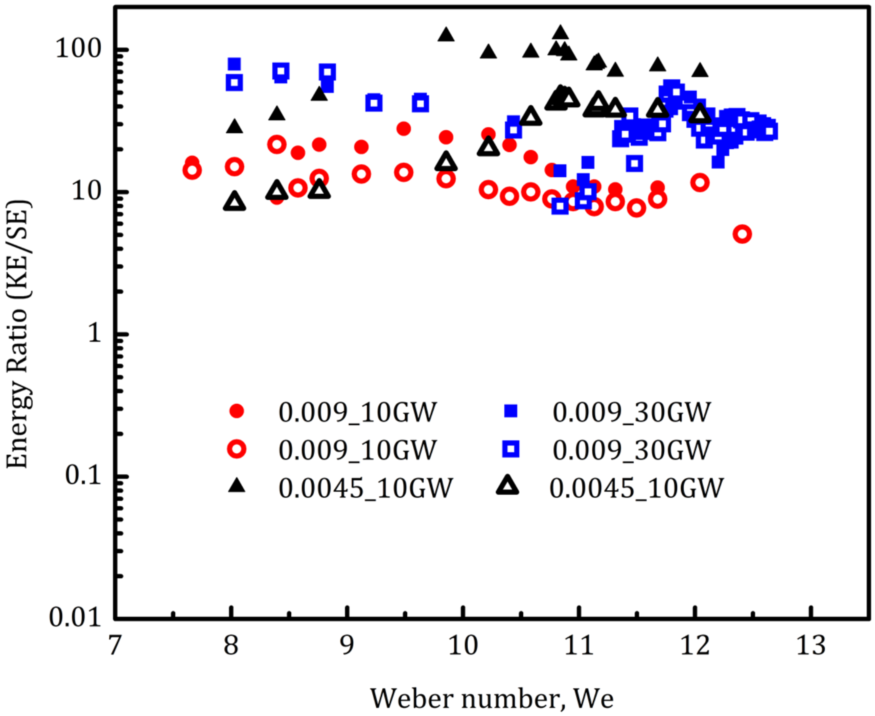

Figure S7: Ratio of kinetic energy to surface energy with Weber number for different pore opening sieves (#0.009, #0.0045) and liquid solution (10% and 30% glycerol water solution). The closed and open symbols represent impact and recoil cavity dynamics, respectively. The Weber number in this study is associated with the impact droplet and is defined as We $\sim\rho R_O V_{imp}^2/\gamma$, where R_O is the impact droplet radius and V_{imp} is the impact droplet velocity.

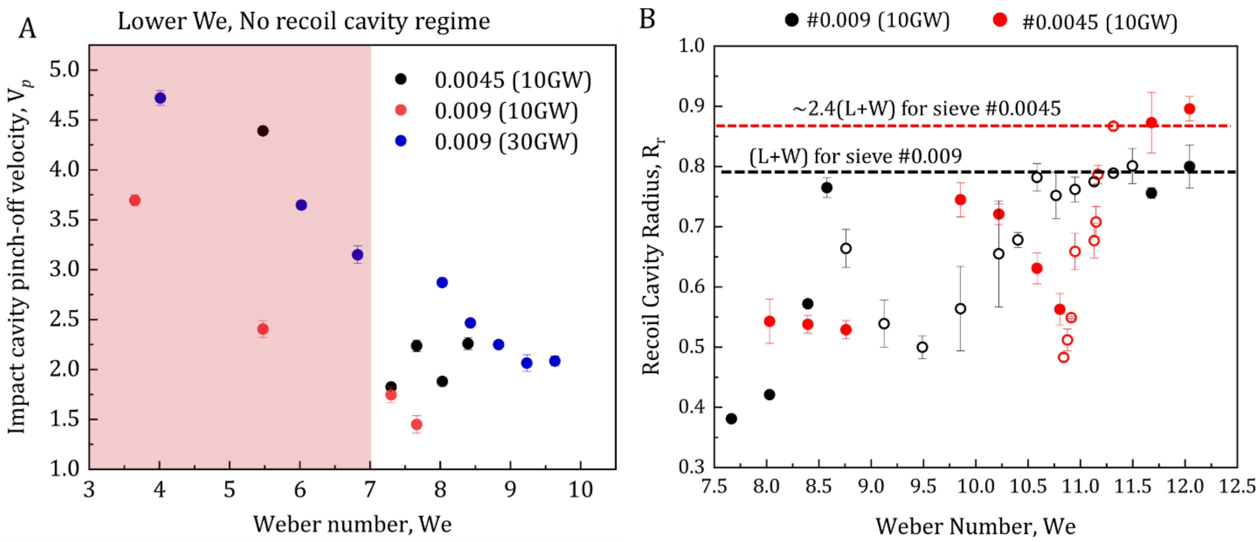

Figure S8: (a) The impact cavity pinch-off velocity plot at low Weber number regime (We < 7) for different sieves and liquids. (b) Recoil radius width versus Weber number for two pore opening sieves (#0.009, 0.0045). The open symbols represent the single drop ejection zone. The multiple droplet regime for sieve #0.0045 is ~ 2($L+W$) whereas for sieve #0.009 it is less than ($L+W$).

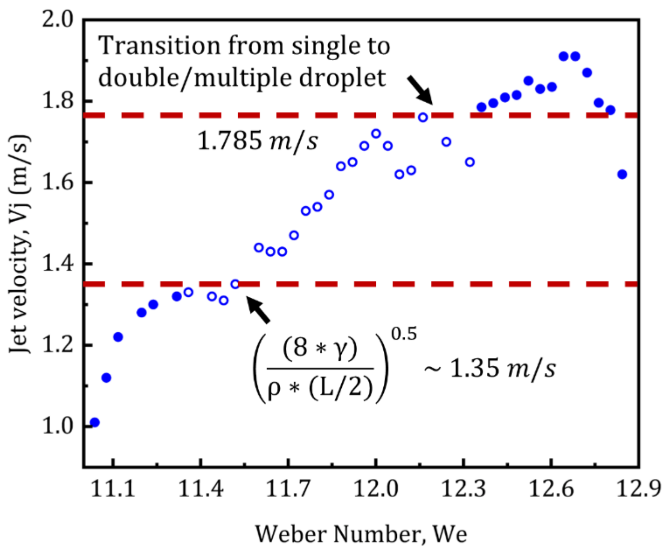

Figure S9: Plot showing ejected jet velocity versus Weber number. The hollow circle shows the single droplet range and its extremes. The lower limit is governed by minimum velocity of droplet ejection model[2], $V_{min} \sim ((8*\gamma)/(\rho*L))^{0.5}$. The upper limit is modeled using theoretical number of droplet (N) ejection model[3], $N \sim ((4*V_j)/(2\pi V_{min}) + 1$. For jet velocity of 1.785m/s and critical threshold velocity 1.35 m/s gives the number of theoretical droplet to 1.82 which is close to 2.

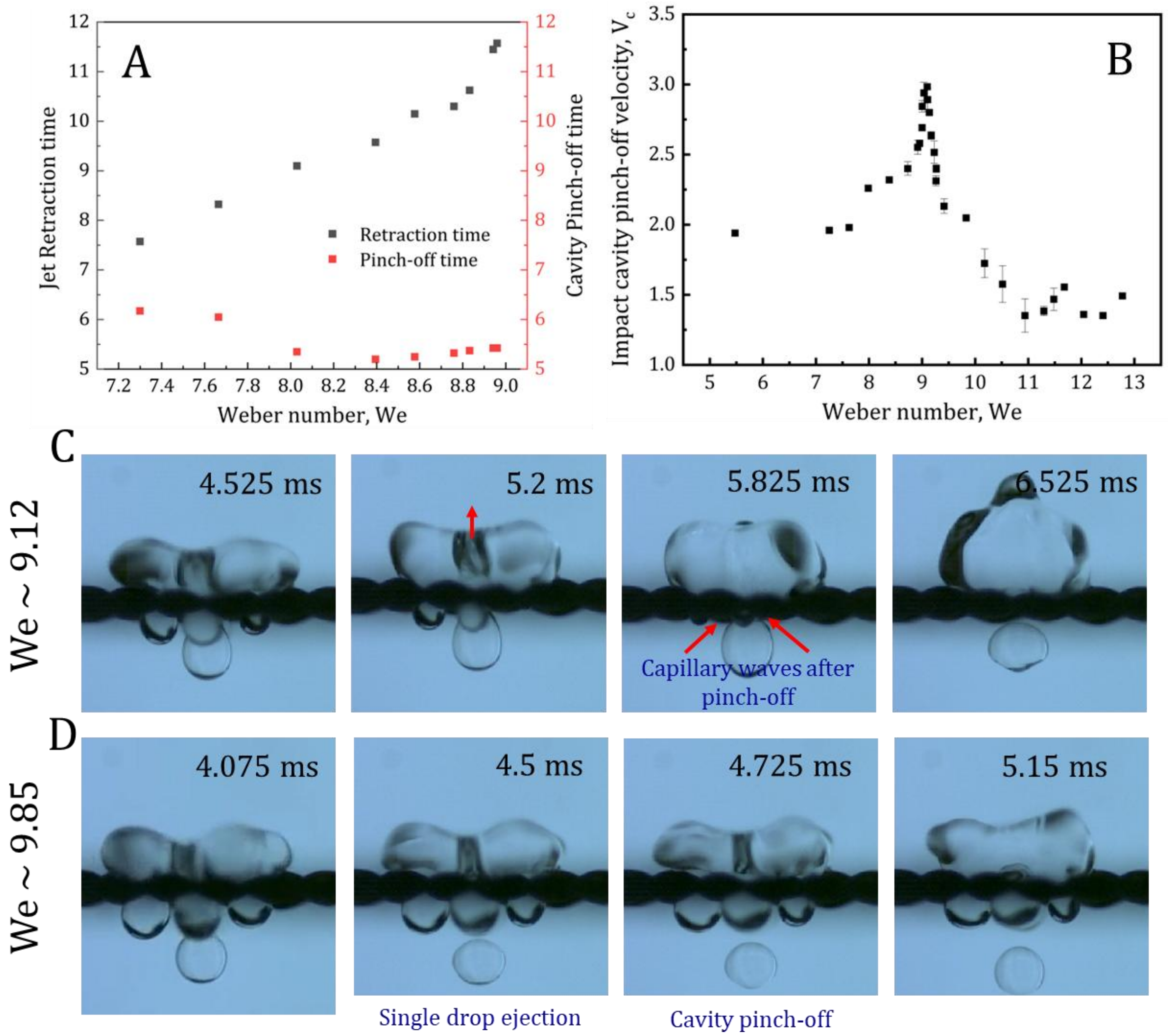

Figure S10: (A) Jet retraction time and Cavity pinch-off time versus Weber number. The time was calculated with reference to when the droplet touches the sieve; (B)Plot showing impact cavity pinch-off velocity versus Weber number. Time-lapse sequence of the superhydrophobic sieve of pore opening 534 μm for different Weber numbers (C) We ~ 9.12; (D) We ~ 9.85. At We ~ 9.12, sudden pressure suction at the pinch-off vicinity causes deceleration leading to single droplet ejection. At We ~ 9.85, the jet ejects a droplet before pinch-off, due to which, after pinch-off recoil cavity (air suction) is visible at the sieve droplet interface.

Captions for supplementary movies

Movie **S1**: Recoil cavity formation after impact cavity pinch-off (We – 7.6 and 10.94).

Movie **S2**: Cavity dynamics in higher pore opening mesh (#0.012, pore opening-534 μm)

References

[1] Z. Q. Yang, Y. S. Tian, S. T. Thoroddsen, **2020**, 1.

[2] P. C. Duineveld, M. M. De Kok, M. Buechel, A. Sempel, K. A. H. Mutsaers, P. Van de Weijer, I. G. J. Camps, T. Van de Biggelaar, J.-E. J. M. Rubingh, E. I. Haskal, in *Organic Light-Emitting Materials and Devices V*, SPIE, **2002**, pp. 59–67.

[3] É. Lorenceau, D. Quéré, *J Colloid Interface Sci* **2003**, *263*, 244.
